# A Deep Nonlinear Subspace Modeling and Reconstruction for Diffusion‐Weighted Imaging Using Denoising Auto‐Encoder

**DOI:** 10.1002/mrm.70496

**Published:** 2026-08-04

**Authors:** Julius Glaser, Zhengguo Tan, Annika Hofmann, Frederik Bernd Laun, Florian Knoll

**Affiliations:** ^1^ Institute of Radiology University Hospital Erlangen, Friedrich‐Alexander‐Universität Erlangen‐Nürnberg Erlangen Germany; ^2^ Michigan Institute for Imaging Technology and Translation (MIITT), Radiology, University of Michigan Ann Arbor Michigan USA; ^3^ Department Artificial Intelligence in Biomedical Engineering (AIBE) Friedrich‐Alexander‐Universität Erlangen‐Nürnberg Erlangen Germany

**Keywords:** deep learning, deep nonlinear subspace, denoising auto‐encoder, diffusion weighted magnetic resonance imaging, joint reconstruction

## Abstract

**Purpose:**

To present a novel, nonlinear subspace modeling and joint k–q‐space reconstruction technique for high‐resolution, multi‐band, multi‐shell diffusion‐weighted imaging (DWI).

**Methods:**

High *b*‐value (> 1000 s/mm^2^), high resolution DWI has the drawback of generally low signal‐to‐noise ratios (SNRs). We present an approach that leverages a denoising autoencoder (DAE) to learn a latent subspace from biophysically simulated diffusion‐weighted signals. The decoder of this network is then used in the forward operator of the image reconstruction process. The decoded latent images are scaled by the $b_{0}$ image, phase is added and processed as regular DWI images with the forward operator for multi‐shot, multicoil, multi‐slice, k–q‐undersampled acquisition schemes. The performance is investigated with a multi‐shell, multi‐direction imaging brain scan and compared to the results of the multiplexed sensitivity‐encoding (MUSE) reconstruction and locally low‐rank (LLR) regularized reconstruction. The results are further validated by a bias and precision analysis of reconstructed fiber directions.

**Results:**

Comparing the reconstructed data using the proposed method shows improved noise suppression compared to MUSE and more details than LLR‐reconstructed images. Specifically, in the higher *b*‐value domain, the reconstruction results show improved detectability of small structures. This bias and precision analysis showed minimal bias introduction, but higher precision with the proposed method.

**Conclusion:**

Our method combines deep learning, latent signal modeling, joint k–q‐space reconstruction, and biophysical simulation for diffusion data and shows strong noise suppression and a high degree of detail in reconstructed diffusion‐weighted images.

## 1 Introduction

As water diffusion is restricted by cell structures in the human body, and especially perpendicularly to the axons in the brain, diffusion‐weighted magnetic resonance imaging (DWI) [1] can provide unique insights into the microstructure of the human brain [2, 3].

High image resolution calls for sampling a large amount of k‐space data. Moreover, obtaining a good angular resolution requires sampling data with many different diffusion encodings in q‐space. These combined calls can considerably prolong the acquisition time. To reduce this time, EPI sequences [4], parallel imaging [5, 6, 7, 8], and simultaneous multi‐slice (SMS) [9, 10, 11] techniques have been developed. To reach sub‐millimeter resolution, multi‐shot interleaved EPI [12] was introduced. The reconstruction of multi‐shot‐DWI using multiplexed sensitivity‐encoding (MUSE) [13] is able to acquire higher in‐plane spatial resolution than single‐shot EPI acquisitions, but these higher resolutions generally lead to a lower SNR. MUSE can run into reconstruction problems in high‐resolution and/or high b‐value DWI due to the low SNR and potentially due to ill‐conditioning of the interim reconstruction of each shot at high acceleration factors, leading to potential artifacts and g‐factor penalty. To address this problem, different denoising schemes have been developed in recent years such as post‐processing techniques based on local principal component analysis (LPCA) [14, 15] and reconstruction‐based techniques that achieve a denoising with locally low‐rank (LLR) regularization, but which suffer from long reconstruction times [16, 17].

Recently, deep‐learning (DL) based auto‐encoder (AE) networks [18] have been studied as regularization functionals [19, 20]. These AEs were trained on simulated noisy data for MRI spectroscopy and diffusion encodings, acting as prior knowledge to constrain the reconstruction. These AEs learn a compact latent representation and the corresponding subspace mapping of their training data in a non‐linear fashion. This mapping can also directly be used in the forward operator of an image reconstruction problem, increasing the model's influence on the reconstructed result compared to using it as a regularizer. This leads to a similar approach as singular value decomposition (SVD) based linear subspace‐modeling and reconstructions [21, 22]. Since the AE's learned mapping is nonlinear, it can lead to a more accurate representation of nonlinear signals. This has already been shown for T2 relaxometry [23], but has not been demonstrated for DWI yet.

In this work, we propose a joint k–q‐space deep‐modeling and reconstruction based on the decoder of a learned denoising auto‐encoder (DAE), trained with simulated diffusion signals. For simulation of diffusion signals, we used either the diffusion tensor (DT) [24] or the ball‐and‐stick (BAS) [25] model. The decoder, which acts as a latent subspace mapping, is then used in the forward operator of the image reconstruction problem for undersampled DWI data. We compare images of individual q‐space directions, colored fractional anisotropy (cFA), and mean kurtosis (MK) to reconstructions obtained with MUSE, MUSE+LPCA denoising, and LLR‐regularization. Furthermore, we perform a bootstrapping experiment to investigate potential bias introduction for the proposed reconstruction compared to conventional methods.

## 2 Methods

### 2.1 Diffusion Models

In the diffusion tensor model, the measured DWI signal in each image voxel is given by:

(1) $$\frac{S\left(b,g\right)}{S\left(0\right)}=\exp\left(-b\cdot g^{T}\bar{D}g\right),$$

where S(b,g) is the signal loss depending on the diffusion weighting *b*, and diffusion encoding gradients direction ***g***. S(0) is the signal without any diffusion weighting, and $\bar{D}$ is the diffusion tensor.

The DT model assumes a single compartment in each voxel and does not account for crossing fiber regions. This limitation may be overcome with more complex multi‐compartment models such as the ball‐and‐stick model (BAS) [25]. It assumes the presence of one ball compartment and *N* zero radius stick compartments:

(2) $$\frac{S\left(b,g\right)}{S\left(0\right)}=\left(1-\sum_{i=1}^{N}f_{i}\right)\exp\left(-\mathrm{bd}\right)+\sum_{i=1}^{N}f_{i}\cdot \exp\left(-\mathrm{bd}\cdot g^{T}R_{i}AR_{i}^{T}g\right)$$

Here, *N* is the number of sticks, $f_{i}$ the signal fraction of stick *i*, *d* the diffusivity of the ball compartment, and $R_{i}AR_{i}^{T}$ describes the anisotropic diffusion tensor along the stick orientation $\left(\theta_{i},\varphi_{i}\right).$ A is a fixed matrix:

(3) $$A=\left[\begin{array}{lll} 1 & 0 & 0\\ 0 & 0 & 0\\ 0 & 0 & 0 \end{array}\right]$$

and the rotation matrix $R_{i}$ rotates ***A*** to ($\theta_{i}$, $\varphi_{i}$).

### 2.2 DAE Training

**FIGURE 1:**
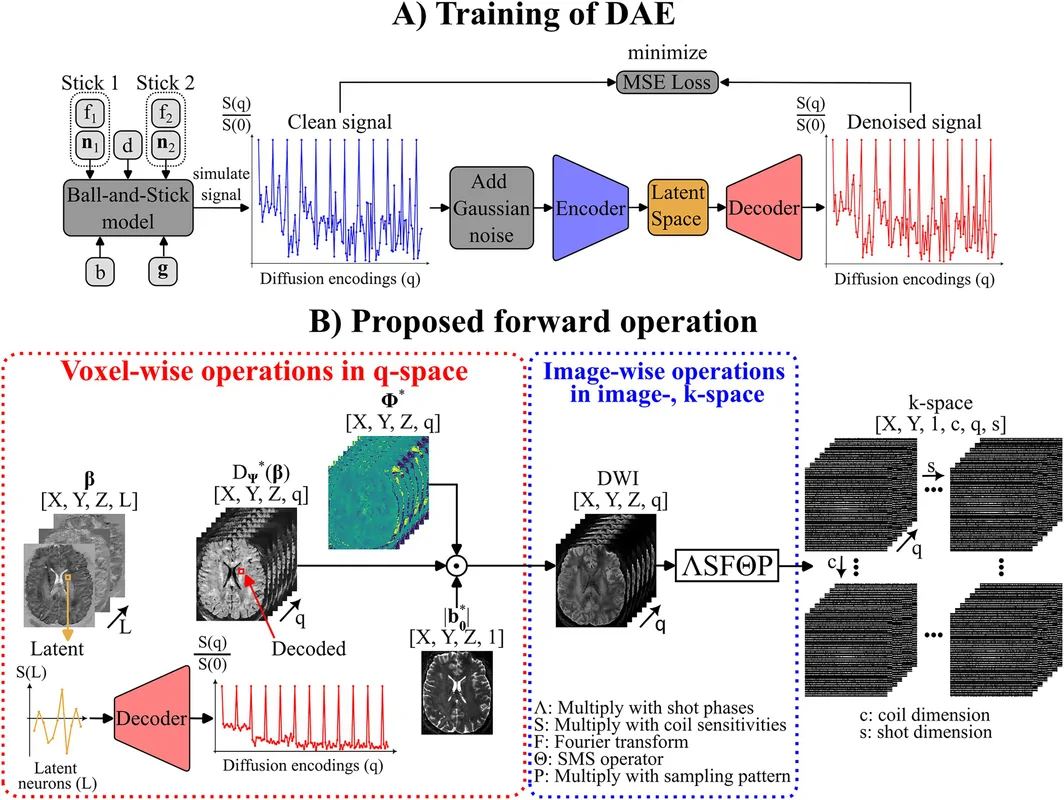
(A) Depiction of the training process of the DAE model and (B) the forward operator of the proposed reconstruction scheme. For the training operation with the BAS simulation model, normalized signals are simulated using randomly drawn and sequence‐dependent parameters. Here *f*_1_ is the fraction of stick 1 and $R_{1}$ its orientation, *d* is the diffusivity, *b* is the *b*‐value used in the sequence for the specific gradient direction, and ***g*** the diffusion encoding gradient direction. Then, Gaussian noise is added to the simulated signal and is used as input to the DAE, which learns to denoise the signal. The loss, which is minimized, is then calculated by the MSE between input and output. The forward operator of our proposed method begins with latent images β of a reduced latent dimensionality L, which are reconstructed using the decoder of the trained DAE. The signal of each voxel across the L dimension is decoded to the full q‐space size, resulting in normalized, decoded q‐space magnitude images. These are then voxel‐wise multiplied with the magnitude of the $b_{0}^{*}$ image and phase $\Phi^{*}$ is added. The resulting DWI data is then used for the remaining multi‐shot, multi‐coil, Fourier transform, SMS and undersampling operations of the acquisition process in an image‐wise manner resulting in the acquired k‐space.

The idea of the training of the DAE can formally be described as follows:

(4) $$\Omega^{*},\Psi^{*}=\underset{\;\Omega ,\;\Psi }{\text{argmin}}{\left\|y-D_{\Psi }\left(E_{\Omega }\left(y+\eta \right)\right)\right\|}_{2}^{2}$$

where $E_{\Omega }$ is the encoder with weights and biases Ω, $D_{\Psi }$ the decoder with weights and biases Ψ of the network, ***y*** a signal curve and η added noise. Data dictionaries with simulated signal curves were used for training. For the simulation, the two presented diffusion models (Equations 2 and 3) were utilized. For BAS, two sticks were considered to resolve for crossing fibers. Details on simulation, network, and training parameters are summarized in Tables S1 and S2. The training process is depicted in Figure 1A. The trained decoder with optimized and fixed weights will be referenced as $D_{\Psi^{*}}$ in the following.

The DAE is built of fully‐connected layers; input and output size was 126 (full q‐space size); the latent‐space size was 11. First and last network layers are fixed to mask S(0) entries of q‐space to have a fixed output of one, corresponding to the S(0) signal. Trainings were performed on an AMD EPYC 7502 (“Rome”, “Zen2”), 64 cores/128 threads CPU. Training time for both DT and BAS models was 4 h. The training was implemented in PyTorch [26] with the usage of DiPy [27]. For ablation studies on network and training parameters see Figures S1–S4.

### 2.3 In Vivo Datasets

Two 3‐shell datasets from two volunteers, one low‐resolution (LR), minimally accelerated reference dataset with two averages and a high‐resolution (HR), highly accelerated dataset were acquired on a clinical 7T MR system (MAGNETOM Terra.X, Siemens Healthineers, Erlangen, Germany). It was equipped with a 32‐channel head coil (Nova Medical, Wilmington, MA, USA) and the XR gradient system (maximum gradient strength 80 mT/m with a peak slew rate of 200 T/m/s). Before the examinations, each subject provided written informed consent and all measurements were approved by the local ethics committee. Sequence parameters are listed in Table 1. Additionally, two more HR and LR datasets with similar parameters were acquired and are evaluated in Figures S16–S25.

**TABLE 1:** Acquisition parameters of the two datasets.

Dataset	#1 (LR)	#2 (HR)
Diffusion mode	Multi‐direction diffusion weighting	
Diffusion scheme	Bipolar^a^	Monopolar
Diffusion direction	114	
*b*‐values (s/mm^2^)	0 (12 repetitions), 1000 (20 directions), 2000 (30), 3000 (64) [28, 29]	
FOV (mm^2^)	200 × 200	220 × 220
In‐plane pixel size (mm^2^)	1.8 × 1.8	1.0 × 1.0
Slice thickness (mm)	2.5	2
Slices	26 (distance factor 50%)	42 (distance factor 20%)
EPI shots	1	2
Averages	2	1
Repetition time (ms)	5200	
Echo time (ms)	91	87
Echo spacing (ms)	0.82	1.00
Bandwidth (Hz/Pixel)	1684	1516
Partial Fourier	6/8	off
Acceleration (in‐plane^b^ × SMS)	1 × 1	3 × 3
Acquisition time (min)	10:58 × 2	23:50

Abbreviations: HR = high resolution; LR = low resolution.

^a^ To reduce distortion artifacts, the minimally accelerated LR dataset was acquired with “bipolar” gradients. “Bipolar” is the vendor specific label for twice‐refocused diffusion‐weightings [30], whereas “monopolar” stands for single‐refocused diffusion‐weightings [31].

^b^ In plane acceleration = 3 means that the acceleration factor was three when combining the k‐space data of both shots. Thus, each individual shot had an acceleration factor of 6 in the HR setting.

### 2.4 Denoising Experiment

To compare the denoising capability of the linear subspace and the DAE based methods, a denoising experiment was performed. For this purpose, additionally to the trained DAEs, an SVD based linear subspace model was implemented, which maps the diffusion signal to a linear subspace equal to the size of the DAE latent‐spaces. Dataset #1 was retrospectively undersampled by only keeping every third sampled k‐space line, SENSE reconstructed without regularization, and as “noisy” magnitude‐data voxel‐wise fed to the three denoising schemes (SVD, DT‐DAE, BAS‐DAE). The data were first normalized by division through the $b_{0}$ image, then denoised voxel‐wise by the three methods and later scaled by the $b_{0}$ signal again. The results were compared qualitatively and quantitatively using the root mean square error (RMSE) to the reference (the fully sampled averaged scan) for three q‐space directions.

### 2.5 Latent‐Space Decoded Reconstruction

For our proposed latent‐space decoded reconstruction, the decoder of the trained DAEs was inserted as the first step of the joint k–q‐space reconstruction operation. This reduces the reconstruction complexity and adds the learned denoising capability of the DAE. However, because the neural‐network only produces a normalized magnitude signal, the decoder output needs to be scaled and combined with phase information. Therefore, the output is multiplied by MUSE pre‐reconstructed $b_{0}$ image magnitude $b_{0}^{*}$ and DWI phase $\Phi^{*}$.

(5) $$b_{0}^{*}=\underset{b_{0}}{\text{argmin}}{\left\|k\left(q=0\right)-Ab_{0}\right\|}_{2}^{2}+\lambda {\left\|b_{0}\right\|}_{2.}^{2}$$

Here $b_{0}\in {\mathbb{C}}^{X\times Y\times Z}$ is the first acquired, SMS‐unfolded $b_{0}$ image with readout dimension *X*, phase encoding dimension *Y*, and the slice dimension *Z*. $k\left(q=0\right)\in {\mathbb{C}}^{X\times Y\times 1\times C\times S}$ is the k‐space data of the first of the q diffusion weightings, the SMS collapsed, multi‐shot (*S* shots), multi‐coil (*C* coils) $b_{0}$ image and A is the forward operator defined as:

(6) A=PΘFSΔ,

where P is the sampling pattern, Θ the SMS operator, F the forward Fourier transform, S the multiplication with coil sensitivity maps, and Δ the multiplication with reconstructed shot‐phases (phase maps were MUSE‐reconstructed from the central k‐space of individual shots and Hanning filtered, see Figure S5). The phase pre‐reconstruction is defined as:

(7) $$\Phi^{*}=\mathrm{Arg}\left(\underset{x}{\text{argmin}}{\left\|k-\mathrm{Ax}\right\|}_{2}^{2}+\lambda {\left\|x\right\|}_{2.}^{2}\right)$$

$\Phi^{*}\in {\mathbb{R}}^{X\times Y\times Z\times q}$ is the phase of the reconstructed complex DWI $x\in {\mathbb{R}}^{X\times Y\times Z\times q}$ and *q* the q‐space dimension.

Using the magnitude of the first of the 12 reconstructed $b_{0}^{*}$ (keeping it optimizable) and $\Phi^{*}$ (held fixed, to prevent optimization issues, due to cross‐dependent gradients during reconstruction) as scaling and the trained decoder additionally to the A operator, the following minimization problem can be formulated:

(8) $$\beta^{*},b_{0}^{**}=\underset{\beta ,{\;b}_{0}^{*}}{\text{argmin}}{\left\|k-A\left[\left|b_{0}^{*}\right|D_{\Psi^{*}}\left(\beta \right)\exp\left(i\Phi^{*}\right)\right]\right\|}_{2}^{2}+\lambda {\left\|\beta \right\|}_{\mathrm{TV}}$$

$\beta \in {\mathbb{R}}^{X\times Y\times Z\times L}$ are the latent images, L the latent‐space size of the trained network, and ${\left\|.\right\|}_{\mathrm{TV}}$ the TV‐norm as regularization function [32], with weighting λ. This was added to spatially smooth the optimized results, reducing in‐plane noise. The forward operator is depicted in Figure 1B.

The optimizations of Equations (5), (7), (8) were done in PyTorch with the stochastic gradient descent (5), (7), and Adam optimizer (8). The influence of $b_{0}^{*}$ finetuning and results of phase pre‐reconstruction can be seen in Figures S6 and S7.

### 2.6 Reconstruction Comparison

To compare the noise suppression of the proposed method, multiple DWI reconstructions of dataset #2 were performed:

1. MUSE reconstruction.
2. MUSE+LPCA denoising
3. LLR‐regularized reconstruction
4. Proposed reconstruction with the DT‐trained DAE (Proposed‐DT)
5. Proposed reconstruction with the BAS‐trained DAE (Proposed‐BAS)

The visual appearance of these high‐resolution images was evaluated and described qualitatively.

Both MUSE and LLR reconstructions were implemented in SigPy [33].^1^ The LLR reconstruction used a block‐shape of (6,6), stride size of (1,1) and a weighting of 0.01, which were adopted from Tan et al. [16]. The low rank criterium was applied across the whole q‐space in image space. Shot‐phases were reconstructed with MUSE. TV‐regularization for the proposed DAE reconstructions was set empirically to λ=0.1.

Diffusion tensors were fitted with *b* = 1000 s/mm^2^ (Shell‐1) data. For this, the proposed reconstruction was rerun only on Shell‐1, because an analysis of a data‐subset is not possible with our joint reconstruction. Additionally, kurtosis [34] fits were calculated for the joint shell reconstructions. All reconstructions were performed on an A100 SXM4/NVLink GPU with 40 GB memory (NVIDIA, Santa Clara, CA, USA). Reconstruction times were: MUSE 1:30 min, MUSE+LPCA 1:45 min, LLR 8 h. The proposed reconstruction was initialized with zeros, run for 150 iterations requiring 2 min, and converged consistently. In Figure S8, a reconstruction using the DAE‐BAS trained network as regularizer (weighting 0.3) together with TV regularization (weighting 0.01) is shown.

### 2.7 Bias and Precision Analysis

For investigation of potential bias in the proposed reconstruction technique, a bootstrapping experiment was performed. For that purpose, the averaged LR dataset #1 was used. The two averages were split, resulting in two independent datasets (LR1 and LR2). These were then retrospectively three‐fold undersampled and reconstructed using parallel imaging SENSE without regularization (PI), PI+LPCA denoising, LLR, and the proposed reconstructions. LLR, Proposed‐DT and Proposed‐BAS hyperparameters were adapted because of the larger voxel‐size (LLR: block‐shape adapted to (3), Proposed: TV‐weight adapted to 0.01). Then 1000 bootstrap samples were created by randomly selecting either the LR1 or LR2 image for every diffusion direction. The evaluation approach was similar to the one presented by Jones [35]. For reference, the fully sampled, averaged, PI dataset was used. fODFs were estimated for all bootstrap samples using multi‐shell multi‐tissue constrained spherical deconvolution [36], implemented in MRtrix3 [37]. From the fODFs, the two main fiber directions were extracted in each voxel. Using the mean dyadic tensor approach [38], bias and precision (95‐th confidence interval) from the bootstrapping experiment for the two main directions in each voxel were calculated. Only fODFs with a peak above an empirically determined threshold were considered for the analysis. The assignment of the two fiber directions obtained in a bootstrap to those of the reference was made by choosing the assignment with the lower difference angle. The bias and precision were evaluated in the whole white matter and in three manually selected areas: Corpus Callosum (CC), Cortico Spinal Tract (CST), and a crossing section (CS) between CC and CST.

Additionally, FA, mean diffusivity, and kurtosis fit results were analyzed for the different reconstructions of retrospectively undersampled LR1 and compared to the averaged reference data.

## 3 Results

### 3.1 DAE vs. SVD Subspace Denoising

**FIGURE 2:**
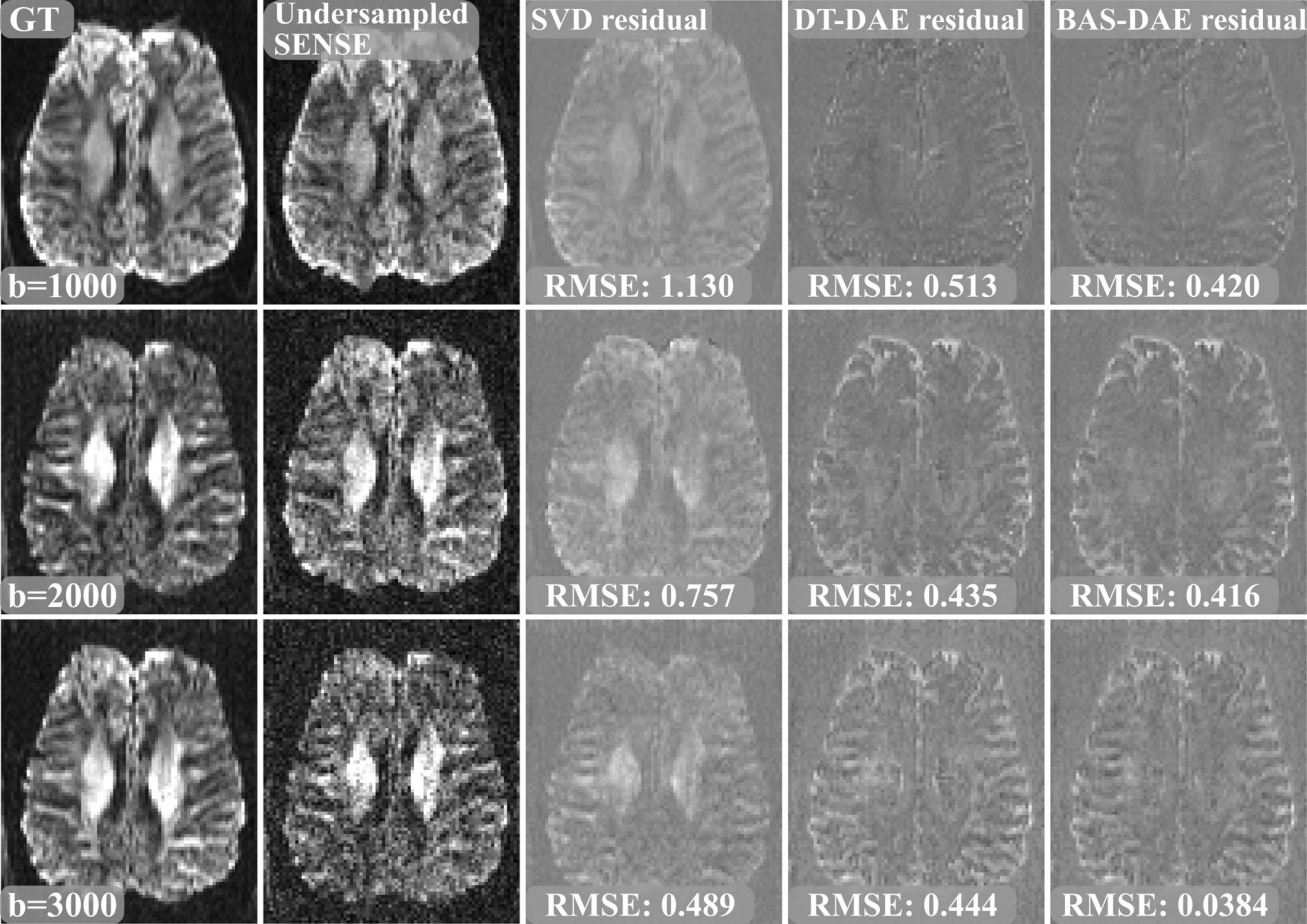
Results of denoising retrospectively undersampled reference data. GT is the low‐resolution, not accelerated data used as ground truth. Undersampled SENSE is the retrospectively undersampled GT (acceleration factor of 3) and SENSE reconstructed images. The residuals are the difference images between the denoising method and the GT. The denoising methods are SVD‐based linear subspace denoising (SVD), DT‐DAE denoising using the DAE trained with the DT simulated data, and BAS‐DAE the denoising with the BAS simulated data. The residuals were scaled up by a factor of 2 for visualization purposes.

Figure 2 shows the results of the denoising experiment with the reference data (GT), the retrospectively undersampled SENSE reconstructed images, and residuals for different *b*‐values (1000, 2000, 3000 s/mm^2^). All methods remove noise from the undersampled SENSE reconstruction. The SVD‐based denoiser introduces a significant bias in terms of image contrast and removal of image details. Both of these effects are reduced with the DL‐based methods. Quantitatively, RMSE is higher for SVD compared to the DAE denoisings. BAS‐trained DAE showed the lowest RMSEs. Visually, only slight differences are visible between the two DAE models.

### 3.2 Reconstruction Comparison

**FIGURE 3:**
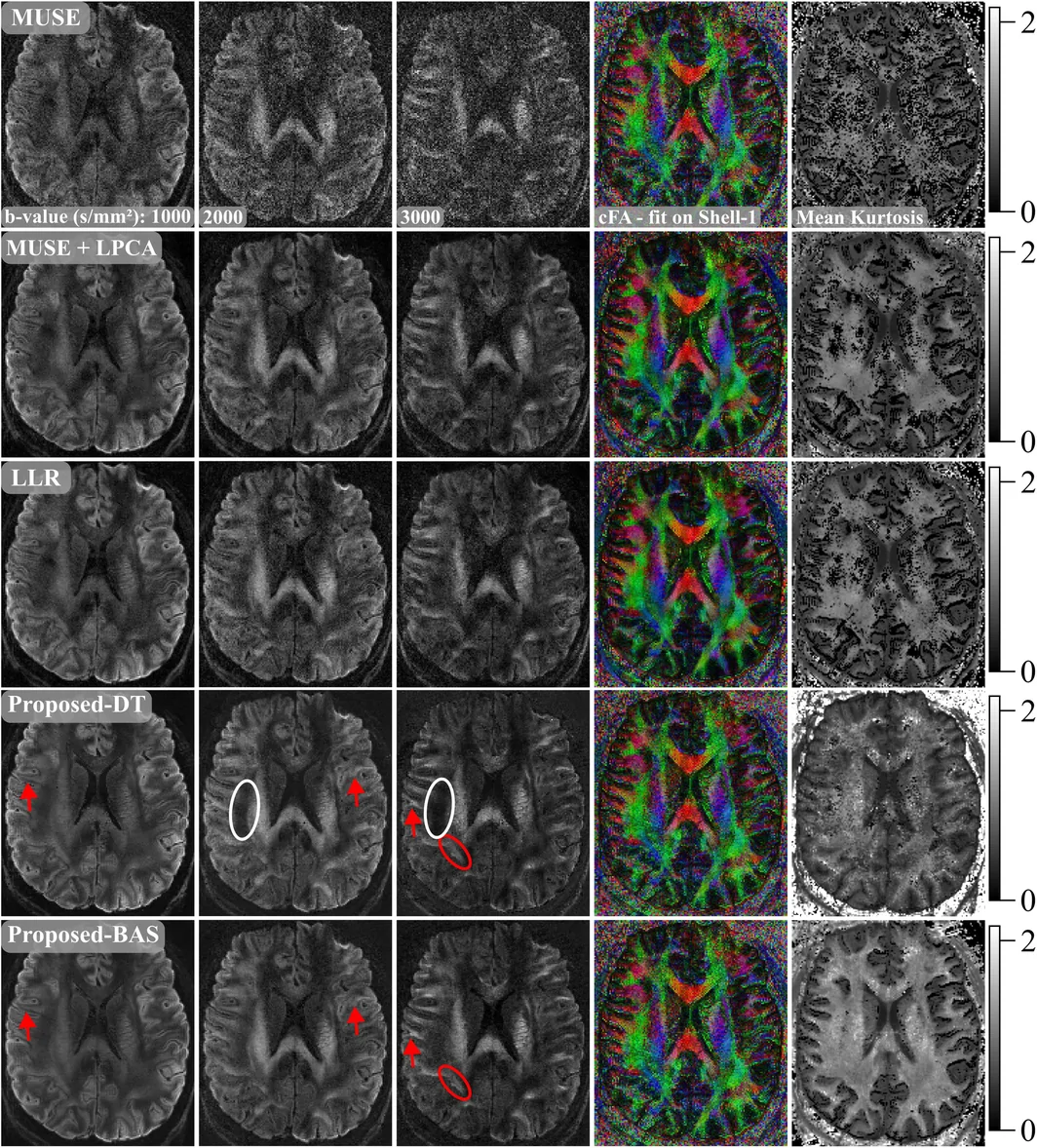
Reconstructed diffusion‐weighted images for one diffusion encoding direction with *b*‐values 1000, 2000, and 3000 s/mm^2^, the resulting cFA maps for a fitting on the *b* = 1000 s/mm^2^ Shell‐1, and the MK fits. The reconstruction techniques are from top to bottom: MUSE, LLR‐regularized reconstruction, LPCA denoised MUSE as well as Proposed‐DT and Proposed‐BAS. The diffusion encoding direction was anterior–posterior for the shown diffusion‐weighted images. U‐fibers are better visible with the Proposed‐DT and Proposed‐BAS methods (red arrows) and some fiber tracts are better preserved in higher b‐values (example: Red ellipse), but for Proposed‐DT the contrast changes for b‐values above 1000 (white ellipse). The cFA on Shell‐1 looks smoother for MUSE+LPCA and LLR. The MK looks noisy and instable for MUSE, misses patches in MUSE+LPCA and LLR, has a different contrast and looks stable in Proposed‐DT and Proposed‐BAS, but with slightly elevated contrast.

The reconstruction results are depicted in Figure 3 for the HR dataset. MUSE reconstructions show substantial noise corruption that increases with the *b*‐value. MUSE+LPCA and LLR reconstructions show a higher SNR than MUSE for all three depicted b‐values at the cost of blurring. The proposed reconstructions show more details and higher SNR for the depicted *b*‐values compared to the other reconstructions. Some contrast differences between DT and BAS images are noticeable.

The cFA maps exhibited the best for LLR and MUSE+LPCA, proposed reconstructions and MUSE showing rather noisy results. The MK images look noisy for MUSE; patches are missing in MUSE+LPCA and LLR; contrast seems different for proposed‐DT. Proposed‐BAS does not show black patches but a slight shift in contrast compared to MUSE+LPCA and LLR. Axial and radial kurtosis can be found in Figure S9. Reconstruction results for DAE‐BAS regularized reconstruction can be found in Figure S8, which shows artifacts and contrast shift in the chosen configuration.

### 3.3 Bias and Precision Analysis

**FIGURE 4:**
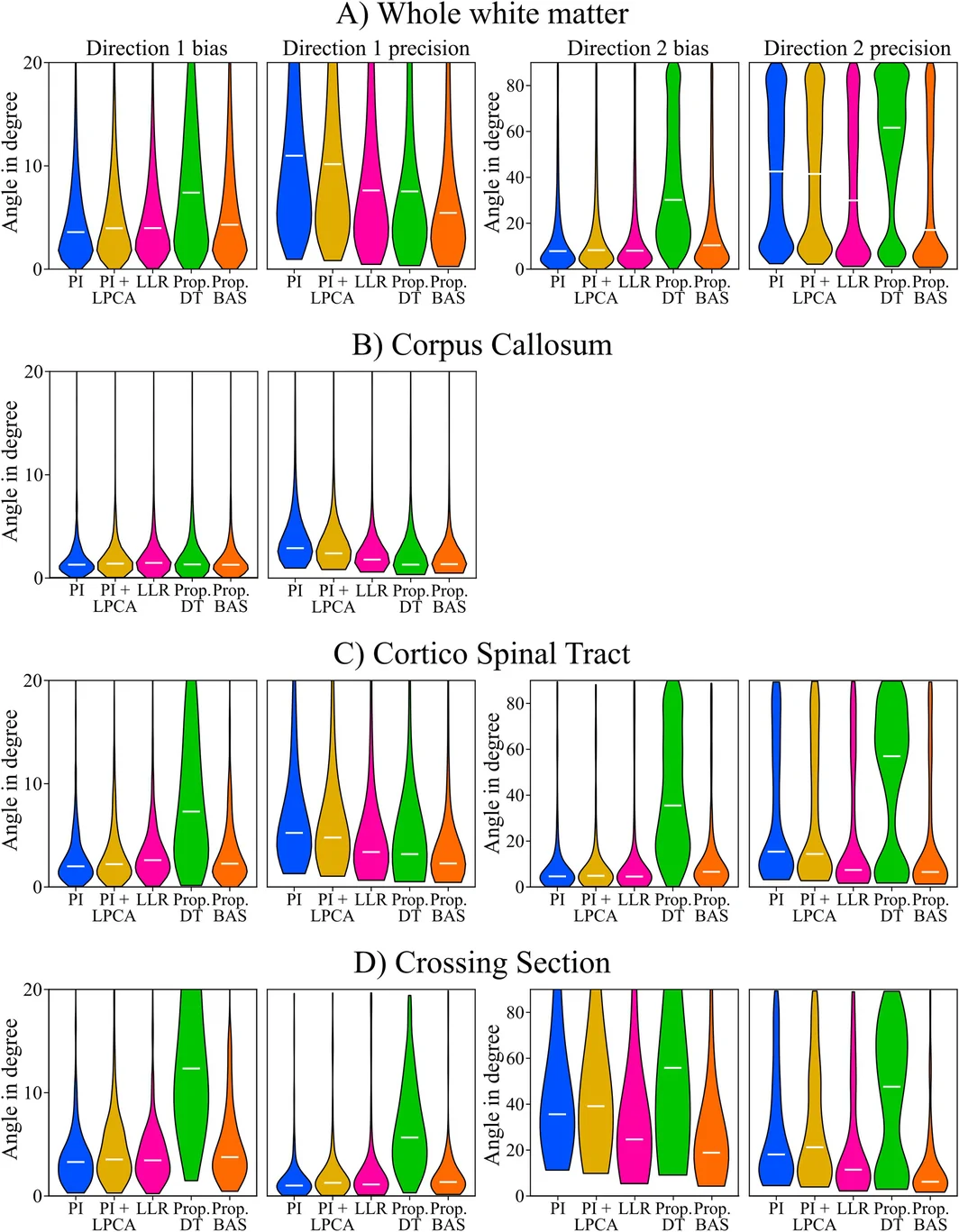
Violin plots describing the bias and precision for main fiber direction 1 and 2 in the areas “whole white matter” (A), CC (B), CST (C), and CS (D) for the reconstructed undersampled data using PI, PI+LPCA denoising, LLR‐regularized reconstruction, Proposed‐DT and Proposed‐BAS. The white line in each violin indicates the median. The CC results show only bias and precision for one direction, as there is only a small number of voxels containing more than one relevant fiber direction.

Figure 4 shows violin plot results for the bias and precision for direction 1 and direction 2 for the white matter (A), CC (B), CST (C), and CS (D). PI overall shows the smallest bias, but all methods perform similarly except proposed‐DT. For precision, Proposed‐BAS shows the best results over all areas and LLR was slightly better than PI and PI+LPCA, while Proposed‐DT performs worst. DAE‐BAS regularized results can be found in Figure S10, which shows no significant improvement for bias and worse precision compared to proposed‐BAS.

Analysis on FA, MD, and kurtosis fits in a histogram‐based approach can be found in Figure S12, Figure S13, and show similar performance for PI, MUSE+LPCA, and LLR. Proposed‐BAS shows slight distribution shifts in radial and axial kurtosis and FA; proposed‐DT shows strong distribution shifts in all metrics, but performs better in FA than proposed‐BAS.

## 4 Discussion

In this work, a novel DWI reconstruction approach was presented that combines aspects of DL, latent signal modeling, joint k–q‐space reconstruction, and biophysical simulation for multi‐shot, high‐resolution DWI reconstruction.

We demonstrated in the denoising experiment that the nonlinear subspace based on DL is superior to the linear subspace in terms of denoising DWI especially at large *b*‐values, showing better results and less bias. We conclude therefore that linear‐subspace modeling is not sufficient for DWI, but DL is, leading to our nonlinear joint reconstruction.

The proposed k–q‐space joint reconstruction shows high noise reduction potential by simultaneously keeping a high degree of structural detail. It outperforms other reconstructions in terms of noise suppression and detail preservation. The proposed method is applicable not only to the acquisition setting presented here. The decoder could be used for other diffusion acquisitions, if the network is trained before on the acquisition. The reconstruction does only require a resolution dependent TV‐weighting. ADAM hyperparameters were kept constant for all reconstructions. The proposed reconstruction performance with the resolution fixed parameters did not vary much across different datasets, while the DAE regularized reconstruction showed a larger performance variation (Figures S15–S24).

We also investigated bias and precision of fODF for the proposed reconstruction and compared it to other established methods and did not observe a concerning bias, but an improvement in precision for the proposed method with BAS training. Proposed‐DT is presumably too simple, however, and showed high bias and low precision in crossing fiber regions. The FA, MD, and kurtosis analysis shows the same effect, as Proposed‐DT introduces a significant shift in higher order metrics as kurtosis, which is not to this extent the case for Proposed‐BAS.

Our proposed method is related to several recent developments in the literature. In the q‐model method by Mani et al. [20], a trained network is used as regularizer. In contrast, in our approach, the trained model is directly included as part of the forward operator, leading to a model‐based reconstruction. This increases the influence of the trained architecture on the reconstruction process, as the results are directly reconstructed from the model and not only loosely guided by it. In this regard, our proposed approach is more closely related to the work by Arefeen et al. [23], who proposed the use of a latent signal model for T2 mapping.

The main limitation of the method, which reduces its performance and applicability, is the capability of the synthetic training data to represent signals observed in in vivo acquisitions. Domain shifts between training and reconstruction may be present. This can potentially lead to biased reconstruction results as it is present in the DT‐trained reconstructions, but also for the BAS model, which is well visible in the distribution shifts in the histogram analysis of the kurtosis fits (Figure S13). Reduction of this domain‐shift is therefore of value for future research. The simulation integrity could be investigated by comparing an increased number of different multi‐compartment diffusion models and their ability to correctly fit real data [39]. The distribution of real data values could be investigated, as the equal distribution over the whole possible parameter spaces could be disadvantageous for the model training, as it could introduce a bias towards unrealistic results. Another limitation is the reconstruction's susceptibility to motion. Images with motion artifacts are forced to align with other images, potentially resulting in artifacts at the outer regions. As the proposed method is voxel‐wise, slight motion can have a stronger effect on the data integrity than patch‐wise methods (e.g., LLR). Using patches in the training process may lead to an improved network architecture and performance, but would increase the training complexity. Larger head movements and substantial eddy current induced image distortions will probably lead to spurious reconstruction results. Addressing these issues is possible [40, 41, 42] and is a topic for future research for the here proposed method. In the data presented in our paper, eddy currents and movement were less of an issue, as young, healthy volunteers were scanned and acceleration was quite high, which also reduced eddy current effects. For the LR data, the bipolar diffusion encoding was used to limit eddy‐current distortions, but it suffers from the need for longer TEs.

Another point of discussion is the value of k–q‐space joint reconstructions in general, as typical metrics such as FA, which are only valid for a subset of the acquired DWI, can potentially not yield the same values as if each q‐space direction is reconstructed separately, and call for a separate reconstruction only on this subset. This effect of “blurring” the q‐space shells, making them lose their independent meaning, is also potentially present in regularization‐based joint reconstructions such as LLR, but may be limited by the weighting of the regularization. Additional analysis of kurtosis fits, mean diffusivity, and FA fits also show this shell blurring behavior, and improvement if the proposed reconstruction is applied on each shell separately (shell‐split reconstruction). However, when using all data for the image reconstruction, SNR can be improved, as the data redundancy can be exploited better. This is also visible in the better (more stable) kurtosis fit results for the proposed method in Figure 3 and Figure S9. And, as our bias and precision analysis suggest, this can lead to a higher degree of precision with a minimal increase in bias, which could be advantageous for further DWI analysis such as fiber tracking.

If one wanted to keep the integrity of typical metrics as FA, a reconstruction which focuses more on the separate shells than on all shells would potentially be needed. This, however, limits the noise suppression potential for each shell, as less redundancy is available, which is also visible in the noisy cFA plots for the Shell‐1 reconstructions of the proposed methods. Another approach would be a direct model‐based reconstruction, which outputs the metrics like FA or MK directly [43]. While this approach still suffers from underlying models, clinically important metrics could be more reliant and stable in generation. This could potentially be adapted to the proposed method by introducing FA and kurtosis metrics in the training process and is of interest for future research. A self‐supervised, zero‐shot training [44, 45] of the proposed DAE‐decoded reconstruction, without an underlying model, could also solve the issue of the domain shift.

## 5 Conclusion

The proposed DWI joint k–q‐space reconstruction for multi‐shot, high‐resolution data leverages the decoder of a DAE in the reconstruction forward operator. The decoder enables a nonlinear subspace mapping and denoising in the DWI reconstruction and leads to significant noise suppression, while retaining sharp structural details.

## Funding

Funding by the German Research Foundation (DFG) is gratefully acknowledged (projects 513220538, 512819079; and project 500888779 of the RU5534 MR biosignatures at UHF). In addition, we gratefully acknowledge the scientific support and HPC resources provided by the Erlangen National High Performance Computing Center (NHR@FAU) of Friedrich‐Alexander‐University Erlangen‐Nürnberg (FAU) under the NHR project b143dc. NHR funding is provided by federal and Bavarian state authorities. NHR@FAU hardware is partially funded by the German Research Foundation (DFG)‐440719683.

## Conflicts of Interest

F.K. receives patent royalties for deep learning image reconstruction and research support from Siemens Healthineers AG, has stock options from Subtle Medical, and is a consultant for Imaginostics.

## Supporting information


**Table S1–S2:** Simulation, network, and training details.
**Figure S1–S4:** Network ablation studies.
**Figure S5:** MUSE shot‐phase estimation.
**Figure S6–S7:** Influence of b0 tuning and images of pre‐reconstructed phase.
**Figure S8:** DAE regularized results of data #2.
**Figure S9:** Radial and axial kurtosis results of data #2.
**Figure S10:** Bias and precision analysis including DAE regularized reconstruction.
**Figure S11–S13:** Reconstruction of retrospective undersampled data #1 and kurtosis and FA metric analysis.
**Figure S14:** Bias and precision angle maps.
**Figure S15:** SENSE reconstruction of individual shots.
**Figure S16–S20:** Results for additional volunteer 3.
**Figure S21–S25:** Results for additional volunteer 4.

## Data Availability

The data that support the findings of this study are available on request from the corresponding author. The data are not publicly available due to privacy or ethical restrictions. Our source code is a publicly available under https://github.com/JuliusGlaser/LASER.

## ENDNOTES

^1^

https://github.com/ZhengguoTan/sigpy.

## References

[1] D. Le Bihan , E. Breton , D. Lallemand , P. Grenier , E. Cabanis , and M. Laval‐Jeantet , “MR Imaging of Intravoxel Incoherent Motions: Application to Diffusion and Perfusion in Neurologic Disorders,” Radiology 161 (1986): 401–407. 3763909 10.1148/radiology.161.2.3763909

[2] C. Pierpaoli , P. Jezzard , P. J. Basser , A. Barnett , and G. Di Chiro , “Diffusion Tensor MR Imaging of the Human Brain,” Radiology 201 (1996): 637–648. 8939209 10.1148/radiology.201.3.8939209

[3] M. A. Horsfield and D. K. Jones , “Applications of Diffusion‐Weighted and Diffusion Tensor MRI to White Matter Diseases—A Review,” NMR in Biomedicine 15 (2002): 570–577. 12489103 10.1002/nbm.787

[4] P. Mansfield , “Multi‐Planar Image‐Formation Using Nmr Spin Echoes,” Journal of Physics C: Solid State Physics 10 (1977): L55–L58.

[5] P. B. Roemer , W. A. Edelstein , C. E. Hayes , S. P. Souza , and O. M. Mueller , “The NMR Phased Array,” Magnetic Resonance in Medicine 16 (1990): 192–225. 2266841 10.1002/mrm.1910160203

[6] J. B. Ra and C. Y. Rim , “Fast Imaging Using Subencoding Data Sets From Multiple Detectors,” Magnetic Resonance in Medicine 30 (1993): 142–145. 8371669 10.1002/mrm.1910300123

[7] K. P. Pruessmann , M. Weiger , M. B. Scheidegger , and P. Boesiger , “SENSE: Sensitivity Encoding for Fast MRI,” Magnetic Resonance in Medicine 42 (1999): 952–962. 10542355

[8] M. A. Griswold , P. M. Jakob , R. M. Heidemann , et al., “Generalized Autocalibrating Partially Parallel Acquisitions (GRAPPA),” Magnetic Resonance in Medicine 47 (2002): 1202–1210. 12111967 10.1002/mrm.10171

[9] A. A. Maudsley , “Multiple‐Line‐Scanning Spin‐Density Imaging,” Journal of Magnetic Resonance 41 (1980): 112–126.

[10] F. A. Breuer , M. Blaimer , M. F. Mueller , et al., “Controlled Aliasing in Volumetric Parallel Imaging (2D CAIPIRINHA),” Magnetic Resonance in Medicine 55 (2006): 549–556. 16408271 10.1002/mrm.20787

[11] K. Setsompop , B. A. Gagoski , J. R. Polimeni , T. Witzel , V. J. Wedeen , and L. L. Wald , “Blipped‐Controlled Aliasing in Parallel Imaging for Simultaneous Multislice Echo Planar Imaging With Reduced g‐Factor Penalty,” Magnetic Resonance in Medicine 67 (2012): 1210–1224. 21858868 10.1002/mrm.23097 PMC3323676

[12] K. Butts , S. J. Riederer , R. L. Ehman , R. M. Thompson , and C. R. Jack , “Interleaved Echo Planar Imaging on a Standard MRI System,” Magnetic Resonance in Medicine 31, no. 1 (1994): 67–72. 8121272 10.1002/mrm.1910310111

[13] N. K. Chen , A. Guidon , H. C. Chang , and A. W. Song , “A Robust Multi‐Shot Scan Strategy for High‐Resolution Diffusion Weighted MRI Enabled by Multiplexed Sensitivity‐Encoding (MUSE),” NeuroImage 72 (2013): 41–47. 23370063 10.1016/j.neuroimage.2013.01.038 PMC3602151

[14] J. V. Manjon , P. Coupe , L. Concha , A. Buades , D. L. Collins , and M. Robles , “Diffusion Weighted Image Denoising Using Overcomplete Local PCA,” PLoS One 8 (2013): e73021. 24019889 10.1371/journal.pone.0073021 PMC3760829

[15] L. Cordero‐Grande , D. Christiaens , J. Hutter , A. N. Price , and J. V. Hajnal , “Complex Diffusion‐Weighted Image Estimation via Matrix Recovery Under General Noise Models,” NeuroImage 200 (2019): 391–404. 31226495 10.1016/j.neuroimage.2019.06.039 PMC6711461

[16] Z. Tan , P. A. Liebig , R. M. Heidemann , F. B. Laun , and F. Knoll , “Accelerated Diffusion‐Weighted Magnetic Resonance Imaging at 7 T: Joint Reconstruction for Shift‐Encoded Navigator‐Based Interleaved Echo Planar Imaging (JETS‐NAViEPI),” Imaging Neuroscience 2 (2024): 1–15. 10.1162/imag_a_00085 PMC12235561 40800457

[17] Y. Hu , X. Wang , Q. Tian , et al., “Multi‐Shot Diffusion‐Weighted MRI Reconstruction With Magnitude‐Based Spatial‐Angular Locally Low‐Rank Regularization (SPA‐LLR),” Magnetic Resonance in Medicine 83 (2020): 1596–1607. 31593337 10.1002/mrm.28025 PMC8720764

[18] G. E. Hinton and R. R. Salakhutdinov , “Reducing the Dimensionality of Data With Neural Networks,” Science 313 (2006): 504–507. 16873662 10.1126/science.1127647

[19] F. Lam , Y. H. Li , and X. Peng , “Constrained Magnetic Resonance Spectroscopic Imaging by Learning Nonlinear Low‐Dimensional Models,” IEEE Transactions on Medical Imaging 39 (2020): 545–555. 31352337 10.1109/TMI.2019.2930586

[20] M. Mani , V. A. Magnotta , and M. Jacob , “qModeL: A Plug‐And‐Play Model‐Based Reconstruction for Highly Accelerated Multi‐Shot Diffusion MRI Using Learned Priors,” Magnetic Resonance in Medicine 86 (2021): 835–851. 33759240 10.1002/mrm.28756 PMC8076086

[21] J. I. Tamir , M. Uecker , W. T. Chen , et al., “Shuffling: Sharp, Multicontrast, Volumetric Fast Spin‐Echo Imaging,” Magnetic Resonance in Medicine 77 (2017): 180–195. 26786745 10.1002/mrm.26102 PMC4990508

[22] F. Y. X. Wang , Z. J. Dong , T. G. Reese , B. Rosen , L. L. Wald , and K. Setsompop , “3D Echo Planar Time‐Resolved Imaging (3D‐EPTI) for Ultrafast Multi‐Parametric Quantitative MRI,” NeuroImage 250 (2022): 118963. 35122969 10.1016/j.neuroimage.2022.118963 PMC8920906

[23] Y. Arefeen , J. S. Xu , M. L. Zhang , et al., “Latent Signal Models: Learning Compact Representations of Signal Evolution for Improved Time‐Resolved, Multi‐Contrast MRI,” Magnetic Resonance in Medicine 90 (2023): 483–501. 37093775 10.1002/mrm.29657 PMC12554331

[24] P. J. Basser , J. Mattiello , and D. LeBihan , “MR Diffusion Tensor Spectroscopy and Imaging,” Biophysical Journal 66 (1994): 259–267. 8130344 10.1016/S0006-3495(94)80775-1 PMC1275686

[25] T. E. Behrens , H. J. Berg , S. Jbabdi , M. F. Rushworth , and M. W. Woolrich , “Probabilistic Diffusion Tractography With Multiple Fibre Orientations: What Can We Gain?,” NeuroImage 34 (2007): 144–155. 17070705 10.1016/j.neuroimage.2006.09.018 PMC7116582

[26] A. Paszke , S. Gross , F. Massa , et al., “Pytorch: An Imperative Style, High‐Performance Deep Learning Library,” Advances in Neural Information Processing Systems, (2019): 32.

[27] E. Garyfallidis , M. Brett , B. Amirbekian , et al., “Dipy, a Library for the Analysis of Diffusion MRI Data,” Frontiers in Neuroinformatics 8 (2014): 8. 24600385 10.3389/fninf.2014.00008 PMC3931231

[28] D. H. Poot , A. J. den Dekker , E. Achten , M. Verhoye , and J. Sijbers , “Optimal Experimental Design for Diffusion Kurtosis Imaging,” IEEE Transactions on Medical Imaging 29 (2010): 819–829. 20199917 10.1109/TMI.2009.2037915

[29] T. Sprenger , J. I. Sperl , B. Fernandez , et al., “Bias and Precision Analysis of Diffusional Kurtosis Imaging for Different Acquisition Schemes,” Magnetic Resonance in Medicine 76 (2016): 1684–1696. 26822349 10.1002/mrm.26008

[30] T. G. Reese , O. Heid , R. M. Weisskoff , and V. J. Wedeen , “Reduction of Eddy‐Current‐Induced Distortion in Diffusion MRI Using a Twice‐Refocused Spin Echo,” Magnetic Resonance in Medicine 49 (2003): 177–182. 12509835 10.1002/mrm.10308

[31] E. O. Stejskal and J. E. Tanner , “Spin Diffusion Measurements: Spin Echoes in the Presence of a Time‐Dependent Field Gradient,” Journal of Chemical Physics 42 (1965): 288–292.

[32] L. I. Rudin , S. Osher , and E. Fatemi , “Nonlinear Total Variation Based Noise Removal Algorithms,” Physica D: Nonlinear Phenomena 60 (1992): 259–268.

[33] F. Ong and M. Lustig , “SigPy: A Python Package for High Performance Iterative Reconstruction,” ISMRM 27th Annual Meeting & Exhibition, 11–16 May 2019 Palais des congrès de Montréal, 1001 Place Jean‐Paul‐Riopelle, Montréal, QC, Canada SMRT 28th Annual Meeting, 10–13 May 2019.

[34] J. H. Jensen , J. A. Helpern , A. Ramani , H. Lu , and K. Kaczynski , “Diffusional Kurtosis Imaging: The Quantification of Non‐Gaussian Water Diffusion by Means of Magnetic Resonance Imaging,” Magnetic Resonance in Medicine 53 (2005): 1432–1440. 15906300 10.1002/mrm.20508

[35] D. K. Jones , “Determining and Visualizing Uncertainty in Estimates of Fiber Orientation From Diffusion Tensor MRI,” Magnetic Resonance in Medicine 49 (2003): 7–12. 12509814 10.1002/mrm.10331

[36] B. Jeurissen , J. D. Tournier , T. Dhollander , A. Connelly , and J. Sijbers , “Multi‐Tissue Constrained Spherical Deconvolution for Improved Analysis of Multi‐Shell Diffusion MRI Data,” NeuroImage 103 (2014): 411–426. 25109526 10.1016/j.neuroimage.2014.07.061

[37] J. D. Tournier , R. Smith , D. Raffelt , et al., “MRtrix3: A Fast, Flexible and Open Software Framework for Medical Image Processing and Visualisation,” NeuroImage 202 (2019): 116137. 31473352 10.1016/j.neuroimage.2019.116137

[38] P. J. Basser and S. Pajevic , “Statistical Artifacts in Diffusion Tensor MRI (DT‐MRI) Caused by Background Noise,” Magnetic Resonance in Medicine 44 (2000): 41–50. 10893520 10.1002/1522-2594(200007)44:1<41::aid-mrm8>3.0.co;2-o

[39] U. Ferizi , T. Schneider , E. Panagiotaki , et al., “A Ranking of Diffusion MRI Compartment Models With In Vivo Human Brain Data,” Magnetic Resonance in Medicine 72 (2014): 1785–1792. 24347370 10.1002/mrm.25080 PMC4278549

[40] W. Wu , P. J. Koopmans , J. L. R. Andersson , and K. L. Miller , “Diffusion Acceleration With Gaussian Process Estimated Reconstruction (DAGER),” Magnetic Resonance in Medicine 82 (2019): 107–125. 30825243 10.1002/mrm.27699 PMC6492188

[41] P. Jezzard , A. S. Barnett , and C. Pierpaoli , “Characterization of and Correction for Eddy Current Artifacts in Echo Planar Diffusion Imaging,” Magnetic Resonance in Medicine 39 (1998): 801–812. 9581612 10.1002/mrm.1910390518

[42] R. Ma , M. Akçakaya , S. Moeller , E. Auerbach , K. Uğurbil , and P.‐F. Van de Moortele , “A Field‐Monitoring‐Based Approach for Correcting Eddy‐Current‐Induced Artifacts of up to the 2nd Spatial Order in Human‐Connectome‐Project‐Style Multiband Diffusion MRI Experiment at 7T: A Pilot Study,” NeuroImage 216 (2020): 116861. 32305565 10.1016/j.neuroimage.2020.116861 PMC7784530

[43] F. Knoll , J. G. Raya , R. O. Halloran , et al., “A Model‐Based Reconstruction for Undersampled Radial Spin‐Echo DTI With Variational Penalties on the Diffusion Tensor,” NMR in Biomedicine 28 (2015): 353–366. 25594167 10.1002/nbm.3258 PMC4339452

[44] J. Cho , Y. Jun , X. Wang , C. Kobayashi , and B. Bilgic , “Improved Multi‐Shot Diffusion‐Weighted Mri With Zero‐Shot Self‐Supervised Learning Reconstruction,” in Medical Image Computing and Computer Assisted Intervention – MICCAI 2023. MICCAI 2023. Lecture Notes in Computer Science (Springer, 2023), 457–466.

[45] Z. Tan , P. A. Liebig , A. Hofmann , F. B. Laun , and F. Knoll , “High‐Resolution Diffusion‐Weighted Imaging With Self‐Gated Self‐Supervised Unrolled Reconstruction,” Magnetic Resonance in Medicine 95 (2026): 2852–2862. 41569109 10.1002/mrm.70250 PMC12962217

