# Supplementary material for: A Deep Nonlinear Subspace Modeling and Reconstruction for Diffusion‐Weighted Imaging Using Denoising Auto‐Encoder
Source: Magn Reson Med. 2026 Aug 4;96(5):2359–69. doi: 10.1002/mrm.70496 (PMC13527252; doi:10.1002/mrm.70496)
Supplement: Supplementary file 1 [file MRM-96-2359-s001.docx]

# Supplemental material

## Simulation, network and training details

For the dictionary simulation, certain parameters were fixed to a value, others were defined in an interval and discretized, all distributions for the discretization were uniform distributions. The diffusivities $d$ were distributed over a biophysical plausible interval of $\left[ 0.1, 3 \right]$ μm²/ms (taken from Jelescu et al. *NMR Biomed*. Jan 2016;29(1):33–47), S(0) was set to one. For BAS, the fractions of the sticks were equally distributed in $f_{i}\in\left[ 0, 1 \right[, while maintaining\sum f_{i}<1$.

The fiber directions were split in principle and secondary direction, where the meaning of the secondary is depending on the model. For BAS the secondary direction is the second sticks direction, for DT the secondary is the second eigenvector of the tensor.

To each of the resulting signals, Gaussian noise was added, representing SNR values between $\left[ 4,\infty\right[$, where SNR is defined as the 1 (S(0)) divided by the standard deviation of the Gaussian noise. This resulted in a total amount of approximately 4.2 million signal curves for DT and BAS. The calculation of the number of signals is the following for DT:

$$N_{samples}=N_{princ}*N_{sec}* \sum_{i=1}^{N_{d}} {i\left( i+1 \right)}/2*N_{noise}$$

$N_{princ}$ being the principal direction, $N_{sec}$ being the secondary direction, $N_{d}$ the number of diffusivities, $N_{noise}$ the number of different applied noise standard deviations. For the BAS dictionary the calculation is the following

$$N_{samples}=N_{princ}*N_{sec}* N_{d}*N_{noise}*\sum_{i=1}^{N_{frac}} i$$

$N_{frac}$ being the number of stick fractions $f_{i}$.

The DAE was trained with 80 % of these signals, 20 % were used for validation.

| Parameter | Value / Interval | N sample points |
| --- | --- | --- |
| S(0) | 1 |  |
| Principal directions | 60 |  |
| Secondary directions | 59 (BAS), 40 (DT) |  |
| d | $\left[ 0.1, 3 \right]$ μm²/ms | 10 |
| $f_{i}$ (subject to $\sum f_{i}<1$) (BAS) | $\left[ 0, 1 \right[$ | 10 |
| Standard distribution of added Gaussian noise | $[0, 0.25]$ | 8 |

***Table S1:*** Parameter distribution for signal curve generation for BAS and DT model.

For network parameters Tanh was used as activation function (for the final decoder layer: sigmoid). The model was trained for 100 epochs using Adam^46^ optimizer with a batch size of 512 and a learning rate of 1e-4. and the encoder layer neuron numbers scale the input size of 126 (q-space size of the sequence in use) linearly down to 11, the decoder is built vice-versa. Training was run for 100 epochs and lasted 4 hours.

| Parameter | Value |
| --- | --- |
| Epochs | 100 |
| Learning rate | 1e-4 |
| Batch size | 512 |
| Encoding layers | 3 |
| Decoding layers | 3 |
| Latent space size | 11 |
| Optimizer | Adam |
| Activation function (final layer) | Tanh (Sigmoid) |

***Table S2***: Network parameters of the proposed trained DAE.

## Ablation studies network architecture

Multiple ablation studies for the network were performed and the performance was tested repeating the bias and precision experiments featured in the main text. First the latent-space size was investigated and the results are visible in figure S1. As the optimal latent-space size was not evident a priori, multiple models with different latent-space sizes were trained, other network parameters were the same as in section 2.2. Their performance in testing with the simulated dataset did not directly relate to their reconstruction performance. The bias and precision analysis were performed for these different models and the results are shown in Fig. S1. These values did not change substantially for latent-space sizes larger than 9. Eventually, we chose a little larger latent-space size of 11.

***Figure S1*** Latent space size ablation. Bias and precision plots for different latent-space sizes for whole white matter.

Network depth (number of encoding and decoding layers) was investigated with an ablation study and results are depicted in figure S2. To analyze the influence of the network depth, multiple models were trained with a latent-space size of 11 for 100 epochs with the simulation data from section 2.2. Their performance in testing of the network did not directly relate to their reconstruction performance. Bias and precision analysis were performed for different models and is visualized in this plot. As the precision and bias values do not change significantly upon depths larger than 2, we agreed eventually to a depth of 4. Higher depths have more trainable parameters and therefore the performance can decrease if the same dataset is used for the same number of epochs, but we would not expect a gain in performance if a network with depth of 6 would be trained even longer

***Figure S2*** Bias and precision plots for different network depths for all white matter.

As the main variable for the size of the simulation dataset is the number of directional samples to simulate diffusion signal curves, we investigated the performance for the bias and precision analysis for models trained with different number of drawn samples. The samples contribute to the size of the training data by a multiplication with N $\times$ (N-1), so the larger the number of samples, the larger the training set. The other training and network parameters were the same as in section 2.2. The resulting bias and precision analysis shows that the potential bias is reduced to a minimum if the dataset is large enough ($\geq$ 50 sample directions), but the training time increases the more samples are drawn, so that sample directions ($\geq$70 upwards) do not justify their use

***Figure S3*** Fiber direction samples ablation study bias and precision analysis

Noise and b0 layer ablation study. Bias and precision plots for all white matter for reference (network used in section 2.2), the network without the b0-layer (which scales the b0 entries to 1 automatically) and the network trained with only clean, noise-free data. While the benefit of the b0-layer is almost invisible in this configuration, the reconstruction definitely benefits from noise during training. The b0-layer still have the benefit of preventing suboptimal final decoding stages, where some b0 entries are not automatically rescaled to 1.

***Figure S4*** Noise and b0 layer ablation study.

## MUSE shot-phase estimation

The estimation of the shot-phase is divided in 2 steps

1. MUSE reconstruction of the k-space center (in our case FOV/4), of the individual shots
2. Hanning filtering of the resulting k-space for denoising

The resulting phase maps of the individual shots are then used in the forward operator of the reconstruction as $\boldsymbol{\Delta}$. Figure S5 visualizes the results after k-space center reconstruction and after filtering and the final shot-phases for b=1000 s/mm² (left two columns) and b=3000 s/mm² (right two columns) representing a high and low SNR case.

***Figure S5*** The shot-to-shot phase estimation is a two-step process to acquire the shot maps which are used in the final $\boldsymbol{\Delta}$ operator. The central region of k-space of the independent shot images are reconstructed (in our case FOV/4), see row 1. The second step is a denoising, which was achieved by Hanning windowing, see row 2. The resulting shot maps are shown in row 3. These are then used in the $\boldsymbol{\Delta}$ operator of the forward operator. Columns 1 and 2 show the results for a b=1000 s/mm² direction and columns 3 and 4 for a b=3000 s/mm² direction, which suffers from the low SNR of the high b-value.

## Influence of b0 tuning and images of pre-reconstructed phase

Figure S6 shows the results for the original pre-reconstructed b0 and the result after being fine-tuned during the proposed reconstruction. This improved quality of the b0 is important for the reconstruction as artifacts in the b0 would propagate into all q-space direction due to the multiplicative dependency in the forward model.

Figure S7 shows the results of the pre-estimated phase. As each direction has an individual phase a finetuning lead in our approaches to either no improvement or suboptimal results, therefore the phase was not finetuned

***Figure S6*** Original, pre-reconstructed b0 ($\boldsymbol{b}_{\boldsymbol{0}}^{\boldsymbol{*}}$**)** vs. fine-tuned b0 after the proposed reconstruction ($\boldsymbol{b}_{\boldsymbol{0}}^{\boldsymbol{**}}$**)**. As the b0 is multiplied onto all directions, a noisy estimate would propagate into all directions and lower the final image quality, therefore a finetuned b0, which was optimized during the proposed reconstruction shows a benefit for final results.

***Figure S7*** Pre-estimated phase $\boldsymbol{\Phi}^{*}$ for three directions representing b-values of 1000, 2000 and 3000 mm²/s. The results are taken directly from the MUSE reconstruction and held fixed, as they are multiplied to all diffusion directions during the proposed reconstruction and if they were optimized as well as the decoder outputs it led to instabilities in the convergence process and worse reconstruction results. As also phase information of the individual direction is carried in the shot phases in the $\boldsymbol{\Delta}$ operator, the influence of these estimated phases was also rather limited, but improved results in the end.

## Results DAE regularized reconstruction

Figure S8 shows reconstruction results of our DAE-regularized reconstruction with also the cFA and kurtosis maps. The reconstruction here is a standard multi-shot, PI reconstruction with additional DAE regularization trained with the BAS model and TV regularization. The reconstruction was implemented in PyTorch and optimized using ADAM with a learning rate of 0.1, the weighting of the DAE regularization was 0.3, the weighting of the TV 0.01.

***Figure S8*** Reconstruction comparison of dataset #2

## Radial and axial kurtosis results

|  | ***Figure S9*** Axial and radial kurtosis results of dataset #2 for MUSE, MUSE and LPCA denoised, LLR regularized reconstruction, proposed reconstruction with DT and with BAS model |
| --- | --- |

## Bias and precision analysis including DAE regularized reconstruction

|  |  |
| --- | --- |
| ***Figure S10*** Violin plots describing the bias and precision for main fiber direction 1 and 2 in the areas “whole white matter”, CC, CST, and CS for the reconstructed undersampled data using PI, PI and LPCA denoising, LLR-regularized reconstruction, Proposed-DT, Proposed-BAS, and here in black the DAE-BAS regularized reconstruction. The white line in each violin indicates the median. The CC results show only bias and precision for one direction, as there is only a small number of voxels containing more than one relevant fiber direction | |

## Reconstruction of retrospective undersampled LR data and kurtosis and FA metrics analysis

|  | ***Figure S11*** Reconstruction comparison for the LR dataset, which are used for the bias and precision analysis in section 2.7. Here, “ground truth” (GT) is the averaged, fully sampled scan, reconstructed with PI. The other reconstructions are based on the retrospectively 3-fold undersampled scan. Proposed-DT and Proposed-BAS are less noisy than the PI images and sharper than the LLR images. DAE-BAS reg is the DAE regularized reconstruction, for the LR data, the weighting was adapted to 0.2 and TV weighting of 0.1. Also, a shell split reconstruction of Proposed-BAS is shown, which help with the underestimation of cFA. cFA fit was done on the b=1000 mm²/s shell. |
| --- | --- |

|  | ***Figure S12*** Kurtosis fits on dataset #1. MK is the mean kurtosis, RK the radial kurtosis, AK the axial kurtosis, MD the mean diffusivity, FA the fractional anisotropy. Fits were done using DiPy for the before mentioned reconstruction methods and a DAE-BAS regularized reconstruction and the proposed reconstruction but where each shell was reconstructed separately. Proposed-BAS under- / overestimates the metrics if joint shell reconstruction is performed, if splitted this effect vanishes mostly. |
| --- | --- |

***Figure S13*** Histogram display of the amplitude of each kurtosis fit metric over all slices of each reconstruction (background samples and invalid fits are ignored) (blue) compared to the GT distribution (red). A certain distribution shift for each metric is to be expected, as the undersampling leads to artifacts in the reconstructions. Generally, the Proposed-BAS reconstruction performs well, except the fractional anisotropy, where it heavily undershoots the expected values, and axial kurtosis shows a clear distribution shift. Proposed-DT has a clearly visible distribution shift for the kurtosis metrics. Splitting the shell reconstruction for proposed-BAS improves the metrics sometimes to a certain degree.

## Bias and precision angle maps

To show that no significant position depended bias or precision is present in Proposed-BAS, we here show the maps, where the angle is color-coded. The bias is similar for all reconstruction for the first direction, but the proposed method using the DT model. The precision seems higher for the proposed reconstruction which also visually show less noise, and highest for Proposed-BAS. For the second angle, the results are similar to the first direction, only Proposed-BAS seems to have a slightly higher bias and the Proposed-DT results are worst by far. The precision seems to be the same as for direction 1 and Proposed-BAS shows the best results, Proposed-DT the worst.

 ***Figure S14*** Bias and precision of the fiber orientation analysis for one slice extracted from the fitted fODFs for the five reconstruction methods PI, PI + MPPCA denoising, LLR-regularized PI, the proposed method with DT and BAS network training.

## SENSE reconstruction of individual shots

To show that even with an acceleration factor of six for each shot (if the shots are combined the acceleration is 3) shows no severe folding artifacts we here show the results for a SENSE reconstruction of each shot and if both shots are combined. Different to the shot-phase estimation reconstructions, these are reconstructed on the full k-space and without any filtering.

***Figure S15:*** Single shot Full FOV reconstructions (acceleration of 6) and with combined shots (acceleration of “3”)

## Results for volunteer 3 (same scan parameters as data #1 and #2 in the main text)

Reconstruction and analysis results from the main text were confirmed by another measurement with another volunteer.

***Figure S16*** reconstruction comparison for a HR dataset for additional volunteer 1. Parameters. Reconstruction parameters were the same as in figure 3 and Figure S5. cFA fit was done on the b=1000 mm²/s shell. cFA fit for the DAE based reconstruction underestimates the FA value. DAE-BAS reg seems to lose the contrast for higher b-values.

|  | ***Figure S17*** Reconstruction comparison for the LR dataset of the additional acquired volunteer 1. Parameters for reconstruction were the same as for Figure S6.  cFA fit was done on the b=1000 mm²/s shell. |
| --- | --- |

***Figure S18*** Bias and precision analysis for LR dataset of additional volunteer 1. Tendencies are the same as for figure 5 in the main text. Only Bas regularized was added and shows worse performance regarding precision for a second fiber in our implementation than the Proposed-BAS.

|  | ***Figure S19*** Kurtosis fits on LR data of additional volunteer 1. Same tendencies as in Figure S7. Proposed-DT fails to get valid results for the Kurtosis fits. |  |
| --- | --- | --- |
|  | | |
| ***Figure S20*** Histogram display of the amplitude of each kurtosis fit metric over all slices of each reconstruction of LR data of additional volunteer 1 (background samples and invalid fits are ignored) (blue) compared to the GT distribution (red). The same analysis as for Figure S8 applies. | | |

## Results for volunteer 4 (same scan parameters as data #1 and #2 in the main text)

Reconstruction and analysis results from the main text were confirmed by one more measurement with another volunteer. FOV was adjusted for this one from 200x200 to 220x220.

***Figure S21*** reconstruction comparison for a HR dataset for additional volunteer 2. Parameters. Reconstruction parameters were the same as in figure 3 and Figure S5. cFA fit for the DAE based reconstruction underestimates the FA value. DAE-BAS reg converges to very noisy solutions for this dataset. The FOV of this volunteer was slightly adjusted. cFA fit was done on the b=1000 mm²/s shell.

|  | ***Figure S22*** Reconstruction comparison for the LR dataset of the additional acquired volunteer 2. Parameters for reconstruction were the same as for Figure S6. The FOV of this volunteer was slightly adjusted for LR data, which might explain differences in reconstruction results. FA fit was done on the b=1000 mm²/s shell. |
| --- | --- |

***Figure S23*** Bias and precision analysis for LR dataset of additional volunteer 2. Values here are a lot higher than for previous reconstructions, which could be due to different acquisition FOV settings, which were not handled in reconstruction. Tendencies stay the same as before.

|  | ***Figure S24*** Kurtosis fits on LR data of additional volunteer 2. Same tendencies as in Figure S7. Proposed-DT fails to get valid results for the Kurtosis fits. |
| --- | --- |

***Figure S25*** Histogram display of the amplitude of each kurtosis fit metric over all slices of each reconstruction of LR data of additional volunteer 2 (background samples and invalid fits are ignored) (blue) compared to the GT distribution (red). The same analysis as for Figure S8 applies.
